# Isolation, identification, and evaluation of lactic acid bacteria with probiotic potential from traditional fermented sour meat

**DOI:** 10.3389/fmicb.2024.1421285

**Published:** 2024-12-12

**Authors:** Jiayi Zhao, Jinshan Zhao, Jinhong Zang, Chuantao Peng, Zhaojie Li, Peng Zhang

**Affiliations:** ^1^College of Food Science and Engineering, Qingdao Agricultural University, Qingdao, China; ^2^Key Laboratory of Special Food Processing (Co-construction by Ministry and Province), Ministry of Agriculture Rural Affairs, Qingdao Agricultural University, Qingdao, China; ^3^Shandong Technology Innovation Center of Special Food, Qingdao, China; ^4^Qingdao Special Food Research Institute, Qingdao, China; ^5^College of Animal Science and Technology, Qingdao Agricultural University, Qingdao, China; ^6^Qingdao JuDaYang Algae Industry Group Co., Ltd., Qingdao, China

**Keywords:** fermented meat, probiotics, molecular identification, antioxidant activity, lactic acid bacteria

## Abstract

Sour meat is a popular traditional fermented product and is a rich source of novel strains with probiotic potential. In this study, we aimed to assess the probiotic potential of lactic acid bacteria (LAB) strains isolated from fermented sour meat. Firstly, the microbial diversity of sour meat from four different areas in China was analyzed. The results showed that LAB were predominant in all samples. Subsequently, LAB were isolated from sour meat and a series of *in vitro* probiotic tests were carried out. A total of 130 bacterial strains with dissolved calcium were obtained and 10 strains showed a range of 89–97% survival in an acidic environment and high tolerance to bile salts. The ranges of hydrophobicity and auto-aggregation of 10 strains were 4.85–80.75% and 1.58–84.2%, respectively. Besides, all 10 strains exhibited high antimicrobial activity and antioxidant activity, of which, DZ24 possessed the strongest free radical scavenging (45.1%) and anti-lipid oxidizing ability (90.3%). Furthermore, DZ24 was identified as *Lactiplantibacillus plantarum* by 16S rRNA gene sequencing. Moreover, the fermentation indexes showed that DZ24 could rapidly reduce the pH to 4.14 and showed high salt and nitrite resistance and antioxidant ability. All the above experimental results indicate that *Lactiplantibacillus plantarum* DZ24 promise a suitable probiotic candidate for future applications in the fermented functional meats.

## 1 Introduction

Fermented foods were parts of the human diet from early civilization and were consumed for thousands of years (Mukherjee et al., 2023). In 2021, Fermented foods were defined as “foods made through desired microbial growth and enzymatic conversions of food components (Mukherjee et al., 2023). They may act as delivery vehicles for probiotics or other “biological” substances, including prebiotics and postbiotics (Long, 2016). Lactic acid bacteria (LAB) are essential microorganisms in fermented foods (Munekata et al., 2021), which can quickly produce acid, inhibit spoilage microorganisms, and ensure food safety (Nie et al., 2023). In addition, these microorganisms are also known for their probiotic effects, such as promoting the absorption of nutrients (Somashekaraiah et al., 2019), improving human immunity (Munekata et al., 2020), and maintaining the balance of intestinal flora (Xing et al., 2016). LAB has also been demonstrated to reduce insulin resistance, relieve oxidative stress, protect *β* cells, and help alleviate diabetes (Wang and Li, 2022). Moreover, some specific LAB strains are capable of producing exopolysaccharides (EPS), which can affect the host by modulating the immune response (Xia et al., 2021). Genera such as *Lactobacillus, Bifidobacterium, Streptococcus, Lactococcus*, and *Pediococcus* are the most common types of probiotic LAB in food (Abid et al., 2019).

Numerous studies have confirmed the probiotic potential of LAB isolated from fermented foods, such as artisanal cheese (Albayrak and Duran, 2021), goat’s milk (Tian et al., 2023), fermented mustard (Chang et al., 2015), rose sauce (Xia et al., 2021). With the increasing concern for food nutrition and health, fermented meat products with functional benefits are increasingly favored by consumers (Ayyash et al., 2019). Using probiotics as meat starters can confer fermented meat products its probiotic properties (Wang et al., 2021). However, the characteristics of fermented meat products, such as high salt concentration, low pH, and low water activity, are more likely to cause the inactivation of probiotics from other sources (Mukherjee et al., 2023). Screening for the probiotic potential strains from meat products can solve this problem (Feng, 2020).

In fact, the separation and screening of LAB in fermented meat products have also been reported. For example, *Lactiplantibacillus* strains with probiotic potential were isolated from traditional fermented fish products (Gupta et al., 2021). *P. pentosaceus* with antioxidant effect were isolated from Harbin red sausage (Chen et al., 2015). However, the above studies are only the results of applied studies of specific probiotic strains in specific fermented meat products. Naturally fermented sour meat is a traditional meat product in the minority areas of southwest China. It is mainly made from fresh pork with seasonings such as rice, glutinous rice, cornstarch, chili peppers, salt and sugar. The natural bacteria present in the fermentation environment are utilized for anaerobic fermentation. It is rich in free amino acids and high nutritional value (Huang et al., 2021). It was found that the fermentation environment (pH = 4–5, anaerobic fermentation) of sour meat was suitable for the growth of LAB (Zhang et al., 2020). However, the research on the probiotic potential of LAB in fermented sour meat products is limited. Therefore, this study aimed to screen the LAB with both fermentation and probiotic potential, and evaluate their probiotic properties *in vitro*. It provides some basis for the development of starter culture for functionally fermented meat products.

## 2 Materials and methods

### 2.1 Sample collection

Four samples were collected from the major sourdough producing markets in China. XM refers to the sample from Libo, Guizhou, which was fermented for 3 months. DZ refers to the sample from Zunyi, Guizhou, which was fermented for 6 months. YZ refers to the sample from Lincang, Yunnan, which was fermented for 12 months. MJ refers to the sample from Lincang, Yunnan, which was fermented for 24 months. They were made using unpasteurized pork and following the traditional process without the addition of commercial starter cultures. Samples were stored at −80°C immediately after collection for subsequent experiments.

### 2.2 Determination of microbial diversity in sour meat

Total genomic DNA was isolated from samples using the Tiangen Plant Genome Kit (Shandong Qingdao Weilai Biology Science and Technology Co., Ltd.) according to the manufacturer’s protocol. DNA quality and quantity were assessed using absorbance ratios of 260–280 nm and 260–230 nm. The DNA was then stored at −80°C until further processing. The V3-V4 region of the bacterial 16S rRNA gene was amplified with primers 338F (5′-ACTCCTACGGGAGGCAGCAG-3′) and 806R (5′-GGACTACHVGGGTWTCTAAT-3′). PCR amplification was performed using a total volume of 50 μL, which contained 5 μL of buffer, 1 μL of Taq Polymerase, 1 μL of dNTP, 10 μM of each primer, and 1 μL of genomic DNA. Thermal cycling conditions were as follows: an initial denaturation at 95°C for 5 min, followed by 35 cycles at 95°C for 1 min, 50°C for 1 min, and 72°C for 1 min, with a final extension at 72°C for 7 min. The PCR products from the first step of PCR were purified using VAHTS DNA Clean Beads. A second-round of PCR was then performed using a total volume of 40 μL that contained 20 μL of 2 × Phusion High-Fidelity Master Mix (New England BioLabs), 8 μL of ddH_2_O, 10 μM of each primer, and 10 μL of PCR products from the first step. Thermal cycling conditions were as follows: an initial denaturation at 98°C for 30 s, followed by 10 cycles at 98°C for 10 s, 65°C for 30 s, and 72°C for 30 s, with a final extension at 72°C for 5 min. Finally, all PCR products were quantified using a Nanodrop™ 2000 spectrophotometer (Thermo Scientific, Wilmington, DE, United States) and pooled together. HTS analysis of bacterial rRNA was performed on the purified, pooled sample using the Illumina Hiseq 2,500 system (San Diego, CA, United States) (2 × 250 paired ends) at Biomarker Technologies Corporation, Beijing, China (Wang et al., 2022).

### 2.3 Isolation of bacterial strains

Isolation of bacterial strains were based on the method described by Grujovic et al. (2022). A total of 5 g samples was weighed and mixed with 45 mL of 0.9% (g/L) sterile saline. After continuous dilution from 10^−1^ to 10^−7^, the bacterial solution was spread onto plates containing MRS agar (Solarbio, Beijing) with 0.3% CaCO_3_ and incubated at 37°C for 48 h. At the end of the culture period, 40 individual colonies of different shapes were isolated from each sample and purified by streaking onto MRS agar. Pure isolated strains were stored in liquid culture using 20% (v/v) glycerol (MACKLIN, Shanghai) at −80°C for long-term preservation. Isolated strains pre-identified by catalase test, Gram’s staining and cell morphology.

### 2.4 Acid tolerance

The pH tolerance of the isolates were determined by counting viable bacteria and slightly modifying the method described by Yalcinkaya and Kilic (2019). The bacterial cells were cultured overnight (24 h) and centrifuged at 10,000 × g and 4°C for 15 min (5810R, Eppendor, Germany). The cells were washed twice with phosphate-buffered saline (PBS) (pH 7.2) and then re-suspended in MRS broth at pH 3.0 (adjusted with 1.0 M hydrochloric acid). After continuous dilution from 10^−4^ to 10^−7^, the bacterial solution were taken at experimental and control groups and incubated on MRS agar medium at 37°C for 48 h to count the total number of viable colonies. Viable counts were detected on MRS agar plates, and the experiment was carried out in triplicate with the duplicate.

### 2.5 Bile salts tolerance

The bile tolerance of the isolates was determined using the method developed by Saboori et al. (2022). The bacterial cells were cultured for 24 h and then centrifuged at 10,000 × g and 4°C for 10 min. They were washed twice with PBS (pH 7.2), re-suspended in MRS broth containing 0.3% bile salts (bovine) (Solarbio, Beijing), and counted after 3 h. The culture without bile salt was considered the control culture. After continuous dilution from 10^−4^ to 10^−7^, the bacterial solution were taken at experimental and control groups and incubated on MRS agar medium at 37°C for 48 h to count the total number of viable colonies. The experiment was performed in triplicate with duplicate analysis.

### 2.6 Antimicrobial activity determination

The antagonistic effect of the isolates on pathogenic bacteria was evaluated using the agar diffusion method (Rabaoui et al., 2023). Isolates were tested against *Escherichia coli* ATCC 25922*, Staphylococcus aureus* ATCC 12600, *Salmonella enterica serovar typhymurium* CICC 21482, and *Listeria monocytogenes* ATCC 19114. The resurgent isolates were inoculated to MRS broth and incubated for 24 h at 37°C under aerobic conditions. Meanwhile, the targeted pathogens were precultured under the same conditions in Luria-Bertani (LB) broth (Solarbio, Beijing). Fresh cultures of the four targeted pathogens (100 μL) were coated on an LB agar plate and dried. Oxford Cups placed on plates were filled with 100 μL of supernatant obtained from centrifugation of isolate cultures. The diameters of inhibition zones were measured and recorded after incubating at 37°C for 24 h under anaerobic conditions. The inhibitory effect was estimated by the width of the inhibition zone and ranked as high (>25 mm, +++), intermediate (13–25 mm, ++), low (1–12 mm,+), and no inhibition (0 mm, −).

### 2.7 Cell surface hydrophobicity assay

To assess the relative surface hydrophobicity of isolates, the affinity of the cells for hydrocarbons was evaluated using xylene, a nonpolar solvent (Altarugio et al., 2018). The overnight cultures were collected (4,000 g for 15 min) and the pelleted cells were washed twice with 5 mL PBS and then re-suspended in the same buffer. The cell concentration was adjusted to 10^8^ cfu/mL. Then, 1 mL of xylene (Tianjin Fuyu reagent) was mixed with 3 mL of cell suspension swirled for 1 min, and left at 25°C for 1 h. The lower aqueous phase was absorbed, and its absorbance was measured at 600 nm in triplicate to calculate the cell surface hydrophobicity (%). The hydrophobicity was evaluated using the following equation:


(1) $$\text{Hydrophobic rate}\;\left(\%\right)=\left(1-\frac{A_{1}}{A_{0}}\right)\times 100\%$$

where A_0_ and A_1_ are the absorbance at 600 nm before and after extraction with xylene, respectively.

### 2.8 Cell auto-aggregation assay

Auto-aggregation of isolates was determined using the method described by Topçu et al. (2020). The culture in MRS broth was centrifuged (8,000 × *g*, 10 min) to obtain a bacterial suspension, washed twice, and resuspended in PBS. Cell concentrations were adjusted to approximately 10^8^ cfu/mL and 3 mL of re-suspended cells were transferred to the test tube and kept at 37°C for 0 and 24 h. Then, 1 mL was taken from the upper part of the cell suspension, and the cell density was determined spectrophotometrically by measurements at 600 nm. The auto-aggregation was evaluated using the following equation:


(2) $$\text{Auto}-\text{aggregation rate}\;\left(\%\right)=\left(1-\frac{A_{1}}{A_{0}}\right)\times 100\%$$

where A_0_ and A_1_ are the absorbance at 600 nm before and after 24 h of incubation.

### 2.9 Antibiotic susceptibility assay

The isolates were evaluated for their antibiotic susceptibility against ampicillin (10 μg), gentamicin (10 μg), vancomycin (30 μg), tetracycline (30 μg), erythromycin (15 μg), and clindamycin (2 μg) antibiotic discs (Changde Beekman Biotechnology Co, Hunan, China) following the method suggested by Nguyen et al. (2023). The bacterial suspension (100 μL) was coated evenly on the surface of the MRS agar plate. Antibiotic discs were placed on the plates and incubated at 37°C for 48 h. Results were expressed by measuring the diameter of the zone of inhibition and interpreted as sensitive (S), resistant (R), and intermediate (I) as per the manufacturer’s protocol.

### 2.10 Hemolytic activity

A safety test for the isolated strain was performed using the method described by Wu et al. (2022). Isolates were inoculated on a blood agar plate and cultured at 37°C for 24 h. The hemolytic activity was described as *α* hemolysis (grass green color zones around the colonies), *β* hemolysis (a clear zone of hydrolysis around the colonies), and *γ* hemolysis (no zone around the colonies). *Staphylococcus aureus* ATCC 12600 (*S. aureus* ATCC 12600) was applied as the positive control.

### 2.11 DPPH radical scavenging assay

The DPPH free radical scavenging ability of isolates was determined by Raman et al. (2022). The culture in the MRS broth was centrifuged (8,000 × *g*, 10 min) to obtain a bacterial suspension, washed twice, and re-suspended in PBS. Bacterial solutions were divided into two groups. One group was crushed with a cell crusher (250 W 10 S/10 s, 10 min), and then centrifuged (9,500 × *g*, 4°C, 15 min) to prepare the supernatant of cell-free culture. DPPH (Shanghai McLean) solution (2 mL) was mixed with both the bacterial solution and the acellular supernatant groups. The mixture was incubated in the dark for 30 min, and the optical density of the mixture was measured at 517 nm (A_1_). Two milliliters of deionized water was mixed with 2 mL of ethanol (95%) as blank group (A_2_); two milliliters of DPPH was mixed with 2 mL of ethanol (95%) as control group (A_3_). The DPPH radical scavenging rate was calculated as follows:


(3) $$\text{DPPH radical scavenging rate}\left(\%\right)=\left[1-\left(\frac{A_{1}-A_{2}}{A_{3}-A_{2}}\right)\right]\times 100\%$$

where A_1_, A_2_, and A_3_ represent the absorbance of the sample, control, and blank groups, respectively.

### 2.12 Superoxide anion radical scavenging activity assay

The activity of the strains were analyzed following the method described by Rwubuzizi (2023). A total of 0.1 mL of the microbial sample was mixed with 4.5 mL of Tris–HCl solution (0.05 mol/L, pH 8.2). The mixture was incubated in a water bath at 25°C for 20 min. Next, 0.4 mL of o-benzyltriol (0.25 mol/L, pre-warmed to 25°C) was added to the mixture, and the reaction was incubated at 25°C for 10 min. The reaction was stopped by adding 0.1 mL of 8 mol/L HCl. To calculate the superoxide anion radical scavenging activity, the absorbance was measured at 320 nm. SASA was calculated as follows:


(4) $$\text{SASA}\left(\%\right)=\left(\frac{A_{0}-A_{1}}{A_{0}}\right)\times 100\%$$

where A_1_ is the absorbance of the sample, and A_0_ is the absorbance of the solution without the sample.

### 2.13 Lipid peroxidation inhibition activity assay

The activity of the strains were analyzed following the method described by Tian and Liu (2018). Twenty milliliters of linoleic acid emulsion included 0.1 mL of linoleic acid, 0.2 mL of Tween 20, and 19.7 mL of deionized water. An aliquot (0.5 mL) of sample (cell suspension or intracellular cell-free extract) was mixed with 1 mL of sodium phosphate buffer (0.02 M, pH 7.4), 1 mL of linoleic acid emulsion and 1 mL of 1% FeSO_4_. After incubation at 37°C for 40 min, the mixture was mixed with 0.2 mL of 4% trichloroacetic acid (TCA) and 2 mL of 0.8% TBA. The reaction was carried out at 100°C for 30 min and cooled. The suspension (1 mL) was mixed with 1 mL of butanol for 1 min, followed by centrifugation at 1800 g for 10 min, and the absorption value at 532 nm (A_S_) was measured. Phosphate buffer was used as a control (A_B_) instead of a sample. This activity can be expressed as follows:


(5) $$\text{Inhibition rate of lipid peroxidation}\left(\%\right)=\left(1-\frac{A_{S}}{A_{B}}\right)\times 100\%$$

where As and A_B_ represent the absorbance of the sample and control groups, respectively.

### 2.14 Bacterial species identification by 16S rDNA sequencing

Total genomic DNA was extracted and purified using a Tiangen Plant Genome Kit (Weilai Biology Science and Technology Co., Ltd., Shandong, China). Bacterial DNA was amplified by PCR using the forward primer 27 F (5′- AGAGTTTGATCCTGGCTCAG −3′) and reverse primer 1,492 R (5′- CTACGGCTACCTTGTTACGA -3′). PCR amplification was carried out in 0.2-mL tubes using PCR amplifier following these steps: pre-denaturation at 95°C for 5 min, denaturation at 95°C for 30 s, renaturation at 58°C for 30 s, elongation at 72°C for 90 s, 35 cycles, with a final extension at 72°C for 7 min. PCR was carried out in 50 μL of reaction mixture, which contained 1 μL of genome LDNA (20 ng/μL), 3 μL of MgCl_2_ (25 mmol/L), 1 μL of deoxynucleotide triphosphate (10 mM), 1 μL of Taq DNA polymerase (5 u/μL), and 1.5 μL of each primer. PCR products were separated by electrophoresis on agarose 1.0% gels. After purification, the samples were sequenced using an ABI3730-XL Genetic Analyzer (Applied Biosystems) (Gupta et al., 2021). A BLAST search was performed to identify sequences deposited in GenBank.

### 2.15 Acid production ability test

The rapid acid production ability of the isolated strains was evaluated using the method described by Murahashi et al. (2020). The culture in the MRS broth was centrifuged (8,000 × *g*, 10 min) to obtain a bacterial suspension, washed twice, and re-suspended in PBS. The isolated strains were inoculated into fresh MRS liquid medium and incubated at 37°C for 0 h (control), 6 h, and 12 h, respectively. The pH was measured using a pH meter.

### 2.16 Nitrite resistance test

According to the national standard, nitrite added to food must not exceed 150 mg/kg. The capability of the strain to grow under fermented meat processing conditions, such as 150 mg/kg sodium nitrite (MACKLIN, Shanghai), in MRS broth, was performed *in vitro* using the method described by Mafra (2020). After 24 h of incubation in MRS broth, the optical density (OD) recorded at *λ* = 600 nm (OD600 nm) was adjusted to 0.1. The mixture was then incubated at 37°C for 0 (control) and 24 h, and the change in absorbance value was measured. All experiments were performed in triplicate.

### 2.17 Amino acid decarboxylase activity test

The activity was analyzed following the method described by Sun et al. (2023). The isolated strains were inoculated into MRS liquid medium containing 10 g/L arginine, lysine, ornithine, and tyrosine (0.6 g/L bromocresol violet as an indicator). A liquid medium without amino acids was used as the control. After incubation at 37°C under anaerobic conditions for 3 d, the color change of amino acid decarboxylase medium was observed. Red or purple color indicated positive, and no color change (yellow) indicated negative (Sun et al., 2023).

### 2.18 Salt resistance test

The level of salt tolerance of isolates was analyzed following the method described by Banik et al. (2023). The isolates were inoculated on MRS agar containing 20, 40, 60, and 80 g/L NaCl at 37°C for 48 h to count the total number of viable colonies. Viable counts were detected on MRS agar plates, and the experiment was carried out in triplicate with the duplicate.

### 2.19 Statistical analysis

All data were expressed as the mean ± standard deviation of three independent measurements. Data were analyzed by the one-way ANOVA after checking for normality by Leven’s test. Duncans multiple comparison tests was used to determine the significant difference (*p* < 0.05) between groups. For data analysis, SPSS software version 19.0 (SPSS Inc., an IBM Company, United States) was used.

## 3 Results and discussion

### 3.1 Diversity of bacterial communities

**Figure 1:**
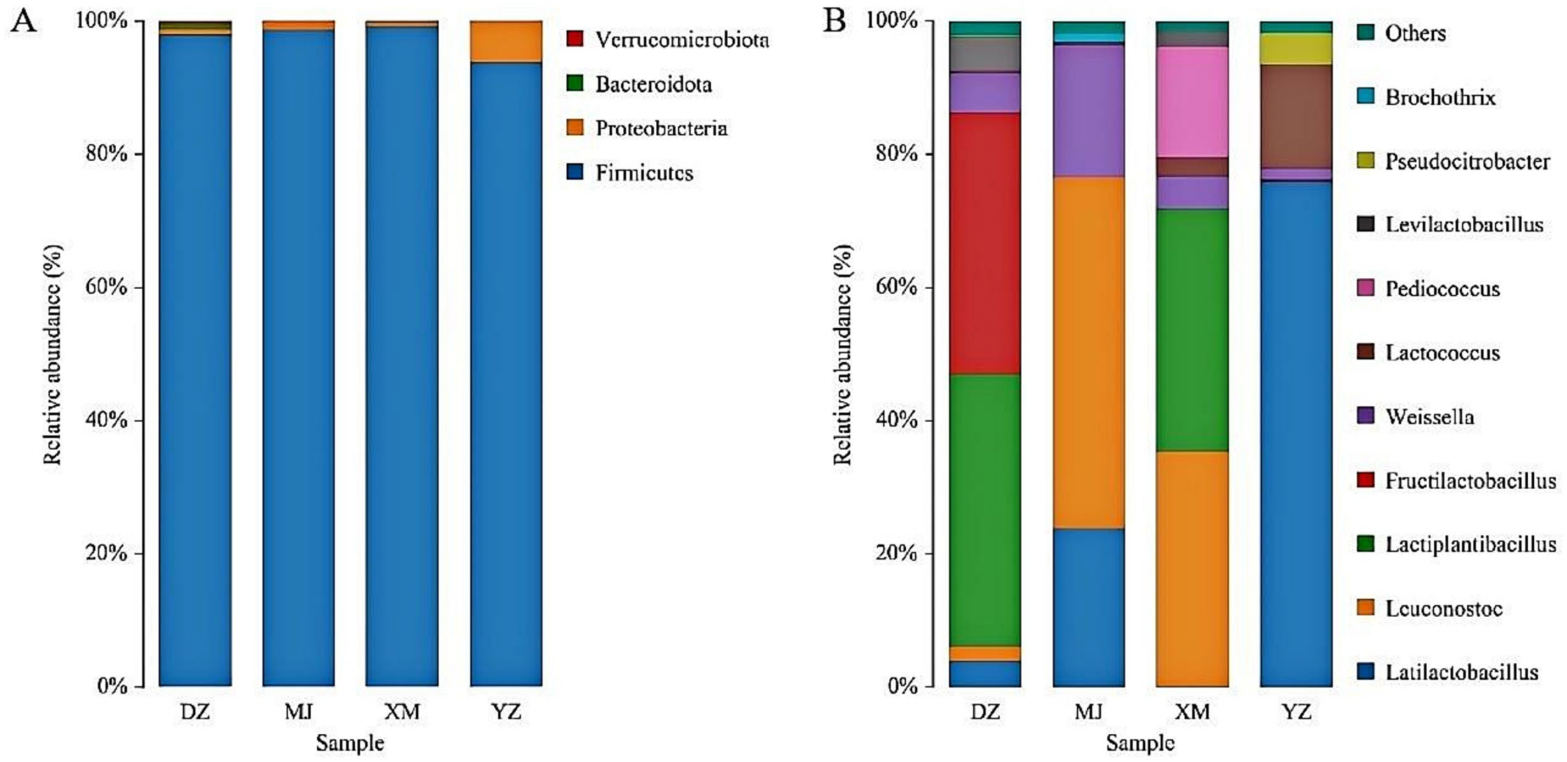
Abundance of the main microbial phylum **(A)** and genera **(B)** found in the different types of Chinese artisanal sour meat. Others represent the relative abundance of all other genera outside the 10 genera **(B)**.

The sequencing data of bacteria was classified at the phylum, and genus, levels to investigate the community structure in depth (Figures 1A,B). The dominant phylum of sour meat were *Firmicutes*, *Proteobacteria* and *Bacteroidota* (Figure 1A)*. Firmicutes* and *proteobacteria* have also been reported to be the dominant flora in sausages and hams (Wang et al., 2021). The distribution of the most abundant genera in the samples was shown in Figure 1B. Samples were dominated by LAB with the main genera being *Latilactobacillus*, *Leuconostoc*, *Lactiplantibacillus*, *Fructilactobacillus* and *Lactococcus*. *Lactobacillus* was reported to comprise the vast majority of the bacterial community of fermented llama meat sausages (Wang et al., 2022). Moreover, the highest abundance of *Lactobacillus* was also found in Suan zuo rou (Wang et al., 2022). This result was consistent with the results of the present study.

### 3.2 Isolation and purification of LAB and resistance to low pH and bile salts

The 127 strains showed more obvious calcium solubilization circles on MRS agar medium, and the colony morphology was white colonies with smooth surface, all of which were Gram-positive bacteria. The colony morphology of some of the strains under oil microscope is shown in Supplementary Figure S1, most of them were bacilli and a few were cocci. The colony counts of different isolates in pH 3 and 0.3% bile salts are presented in Table 1. However, only 10 isolates survived in MRS broth medium at pH 3 and 0.3% bovine bile salts. The resistance values of 10 isolates to low pH and bile salts remained around 7.2–8.2 (log10 CFU/mL) and 4–5.6 (log10 CFU/mL), and the survival rates were 89–97% and 46–72%, respectively. DZ24 showed sufficient viability at pH 3 and bile salt (0.3%) after 3 h, compared with the other isolates. The resistance values of isolates DZ24 was maintained at 8.2 (log10 CFU/mL) and 6.3 (log10 CFU/mL), and the survival rate was 95 and 72%, respectively. The ability to tolerate low pH levels and bile salts is a critical characteristic for probiotics to survive in the unfavorable conditions of the gut (Afshari et al., 2022). It was found that the resistance values of *Lactiplantibacillus plantarum* screened from fermented fish were 6.4–6.6 CFU/mL (Gupta et al., 2021). Besides, the colony number and survival rate of strains isolated from raw milk were 7.4–9.6 CFU/mL, 74–99%, respectively (Zhang et al., 2022). Studies reported that LAB strains isolated from nectar (Somashekaraiah et al., 2019) with probiotic potential were found to have a survival rate above 50% at 0.3% bile. The results were basically consistent with our present study. Therefore, 10 strains performed well *in vitro* tests and were selected for further studies.

**Table 1:** Survival rate of the 14 isolates from sour meat in low pH, bile salts.

Isolates	Viable microorganism count (log cfu/mL)				
Heng	0 h	3 h		Low acid survival rate (%)	Bile salt survival rate (%)
		pH 3.0	0.3 %BS		
DZ24	8.67 ± 0.14^abc^	8.26 ± 0.06^a^	6.31 ± 0.16^a^	95.3	72.8
YZ49	8.15 ± 0.12^d^	7.32 ± 0.10^g^	4.43 ± 0.26^c^	89.8	54.3
XM38	8.85 ± 0.11^a^	8.25 ± 0.10^a^	5.30 ± 0.35^b^	93.2	59.9
XM47	8.45 ± 0.21^bcd^	8.27 ± 0.11^a^	–	97.8	–
DZ52	8.66 ± 0.12^abc^	8.15 ± 0.08^ab^	4.00 ± 0.01^d^	94.1	46.2
XM60	8.86 ± 0.11^ab^	8.23 ± 0.12^ab^	5.59 ± 0.31^b^	92.9	63.1
XM39	8.46 ± 0.14^bcd^	8.10 ± 0.02^abc^	5.52 ± 0.33^b^	95.7	67.9
XM1	8.89 ± 0.22^a^	8.07 ± 0.02^bcd^	5.52 ± 0.08^b^	89.4	64.5
XM42	8.65 ± 0.38^abc^	7.95 ± 0.07^cd^	5.54 ± 0.02^b^	90.5	62.1
XM18	8.65 ± 0.27^abc^	7.80 ± 0.02^de^	–	90.2	–
YZ50	8.17 ± 0.26^d^	7.72 ± 0.18^e^	4.43 ± 0.27^c^	94.5	54.2
XM14	8.41 ± 0.25^cd^	7.75 ± 0.04^e^	–	92.2	–
YZ10	8.35 ± 0.27^de^	7.53 ± 0.17^f^	4.37 ± 0.27^c^	90.2	52.3
MJ112	7.53 ± 0.21^e^	7.26 ± 0.12^g^	–	96.4	–

Each value in the table is the mean ± standard deviation of three experiments each with duplicate. ^a–g^Values in the same row with different superscript letters are significantly different (*p* < 0.05).

### 3.3 Antimicrobial activity

**Figure 2:**
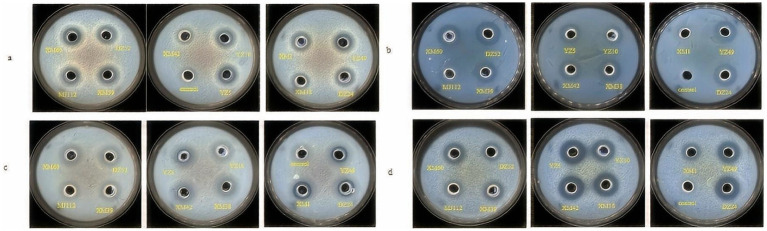
Inhibitory activity of strains against *Escherichia coli* ATCC 25922 **(A)**, *Listeria monocytogenes* ATCC 19114 **(B)**, *Staphylococcus aureus* ATCC 12600 **(C)**, *Salmonella typhimurium* CICC 21482 **(D)**.

The antagonistic effects of the isolates on pathogenic bacteria were shown in Figure 2. XM1 did not inhibit *Listeria monocytogenes*, whereas the isolates showed low-to-moderate antibacterial activity against all pathogens. Isolates was found that they formed an inhibition zone of 10–18 mm on *E. coli*, *S. aureus* ATCC, *Salmonella enterica serovar typhymurium*, and *L. monocytogenes*. Studies was reported that potential probiotic LAB isolated from fermented beverages (Akman et al., 2021) form zones of inhibition 11–20 against *E. coli*, *S. aureus*, *S. typhimurium*, and *L. monocytogenes*, which generally agrees with the results of the present study. As shown in Figure 2, DZ24 has the highest antimicrobial activity (18 mm) for *L. monocytogenese*. Albayrak and Duran (2021) isolated *Enterococcus faecium* MD30 from artisanal white cheese and found an inhibitory (12 mm) effect on *L. monocytogenes*. It has been known that LAB produce organic acids (such as lactic acid) and other compounds that can inhibit bacterial growth and metabolism (Wang et al., 2021). These compounds reduce the intracellular pH of pathogenic bacteria and affect their normal physiological metabolism in the human body to protect gastrointestinal health to a certain extent and reduce gastrointestinal diseases (Wang et al., 2021).

### 3.4 Ability of hydrophobicity and auto-aggregation assays

**Figure 3:**
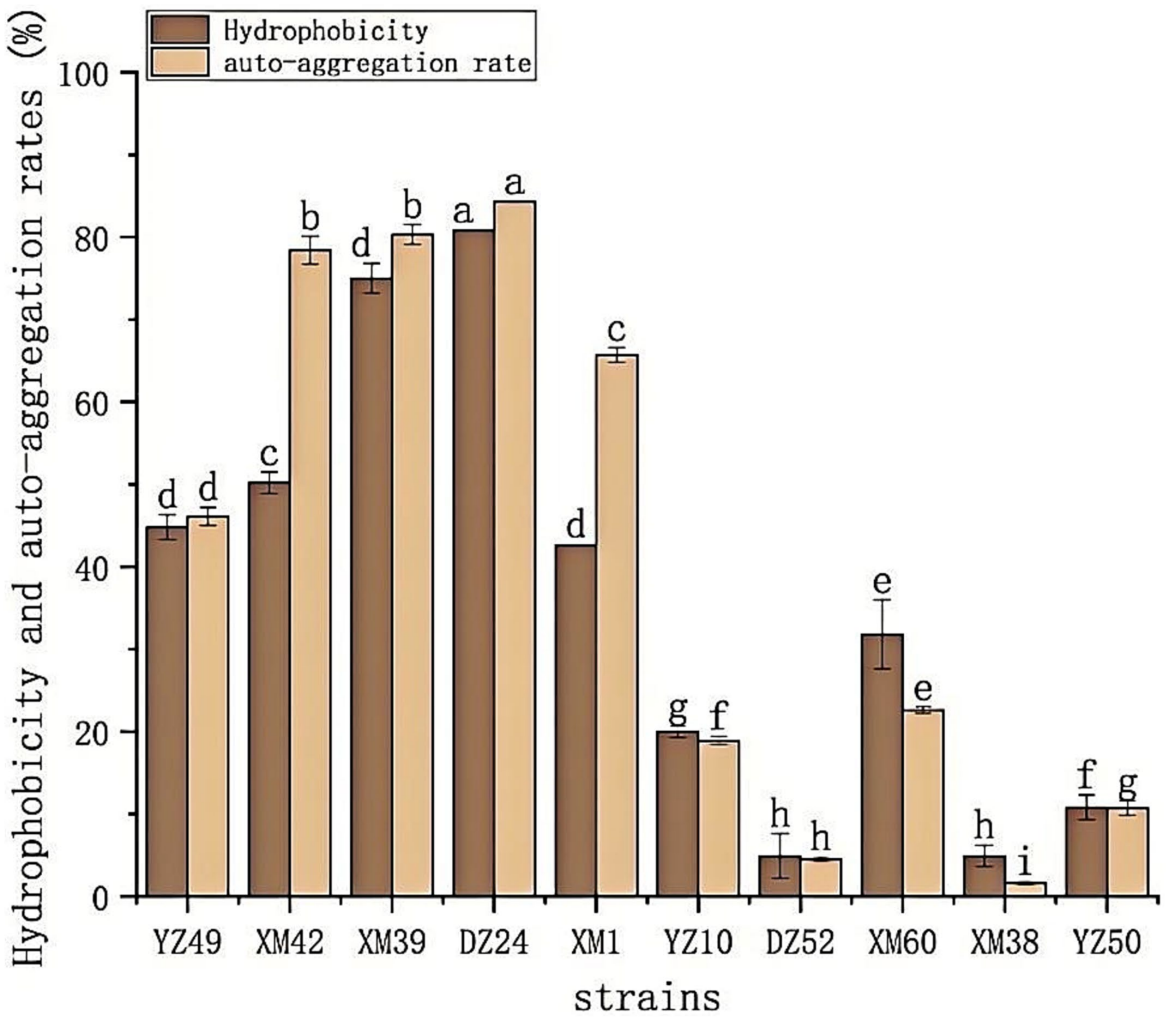
The hydrophobicity and auto-aggregation ability of the 10 isolates. Hydrophobicity and auto-aggregation rate. ^a–g^Values in the same row with different superscript letters are significantly different (*p* < 0.05).

The hydrophobicity and auto-aggregation ability of the 10 isolates are summarized in Figure 3. All 10 isolates of bacteria showed different levels of hydrophobicity and auto-aggregation, ranged from 4.85–80.75% and 1.58–84.2%, respectively. DZ24 exhibited the highest percentage of hydrophobicity (80.75%) and auto-aggregation (84.2%). Recently, the study reported the isolation of *E. durans* MD 57 with good probiotic potential from artisanal white cheese (Albayrak and Duran, 2021), which demonstrated a high hydrophobicity value (41.6%), and the result of this study was much higher than 41.6%. Moreover, previous literatures also established that *Lactobacillus* exhibited a wide range of auto-aggregation of 5–68% (Gupta et al., 2021). Notably, the auto-aggregation rate of DZ24 was 16% higher than in this study. High hydrophobicity makes it easier for bacteria to penetrate the host tissue and exert an effect, indicating that bacteria have good adhesion to the intestinal mucosa and intestinal cells (Raman et al., 2022). The hydrophobicity and auto-aggregation of bacteria are related to their adhesion ability, which is essential for determining bacterial colonization in the intestine (Tang et al., 2022).

### 3.5 Antibiotic susceptibility assay

The safety of the selected isolates was tested based on antibiotic sensitivity, and the results are shown in Table 2. In this study, the results of the proportional calculations showed that the isolates were resistant to vancomycin (100%), tetracycline (90%), erythromycin (50%), ampicillin (10%), gentamicin (80%), and clindamycin (50%). DZ24 was resistant to vancomycin, gentamicin, clindamycin, and erythromycin but sensitive to tetracycline and ampicillin. Sensitivity of LAB to antibiotics is an important criterion for evaluating the safety of potential probiotics (Ozkan et al., 2021). Studies shown that LAB are sensitive to antibiotics (such as erythromycin and tetracycline) that inhibit the synthesis of proteins, but are resistant to aminoglycosides (such as gentamicin) and glycopeptides (vancomycin) (Wu et al., 2022). Moreover, Fan et al. (2022) isolated *Levilactobacillus brevis* from Chinese Bai jiu was resistant to vancomycin, but susceptible to clindamycin. However, according to previous studies, some strains are naturally resistant to common antibiotics, such as vancomycin (Zhang et al., 2020). These studies provide a reference for the experimental results.

**Table 2:** Antibiotic susceptibility and hemolysis test of the 10 isolates in fermented sour meat.

Isolates	VAN	TET	E	AMP	GM	CC	Haemolysis^a^
XM42	R	R	I	S	R	R	γ
DZ24	R	S	I	S	R	R	γ
XM1	R	R	I	S	R	R	γ
XM39	R	R	R	S	R	R	γ
YZ49	R	R	S	R	R	S	γ
XM60	R	I	S	S	R	S	γ
XM38	R	I	S	S	S	R	γ
YZ5	R	I	S	S	R	S	γ
DZ52	R	I	S	S	I	S	γ
YZ10	R	R	S	S	R	S	γ

S, Sensitive; I, Intermediate; R, Resistant; Vancomycin (VAN, 30 μg), Tetracycline (TET, 30 μg), Erythromycin (E, 15 μg), Ampicillin (AMP, 10 μg), Gentamicin (GM, 10 μg), Clindamycin (CC, 10 μg).

a γ-No hemolytic reaction.

### 3.6 Hemolytic reaction

As shown in Table 2 and Supplementary Figure S2, all the isolates had no clear zone on the blood agar plates, surrounding their colonies, and thus were *γ*-hemolytic or non-hemolytic. Some studies reported that the hemolytic activity of LAB with probiotic potential isolated from Duimaj (Soleimani et al., 2023) and homemade pickles (Wu et al., 2022) showed non-hemolytic activity. If hemolysis occurs, the strain may produce hemolysin, which damages body tissues and harms human health (He et al., 2021).

### 3.7 Antioxidant activity

**Figure 4:**
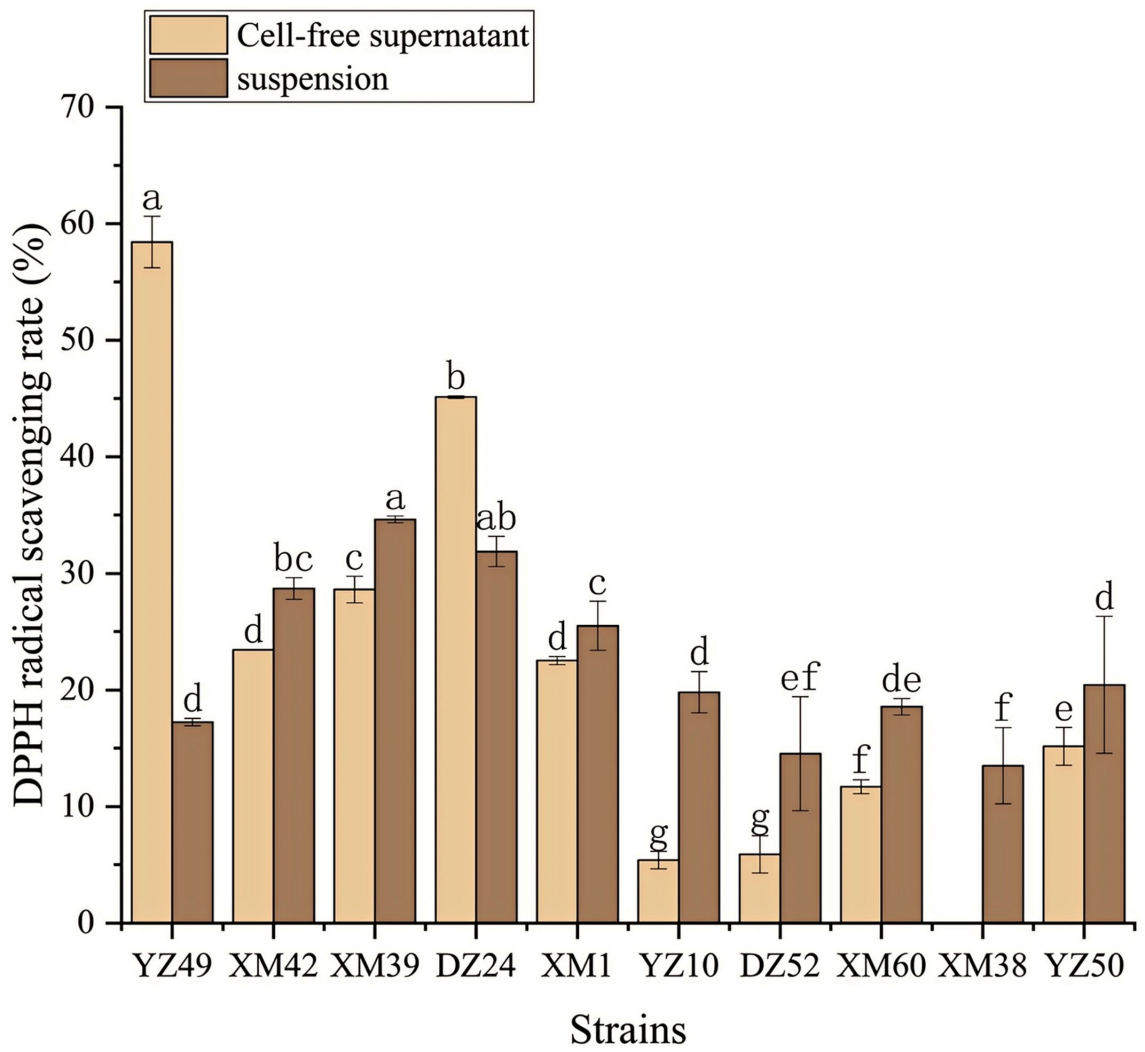
DPPH radical scavenging rate of the 10 isolates. ^a–g^Values in the same row with different superscript letters are significantly different (*p* < 0.05).

**Figure 5:**
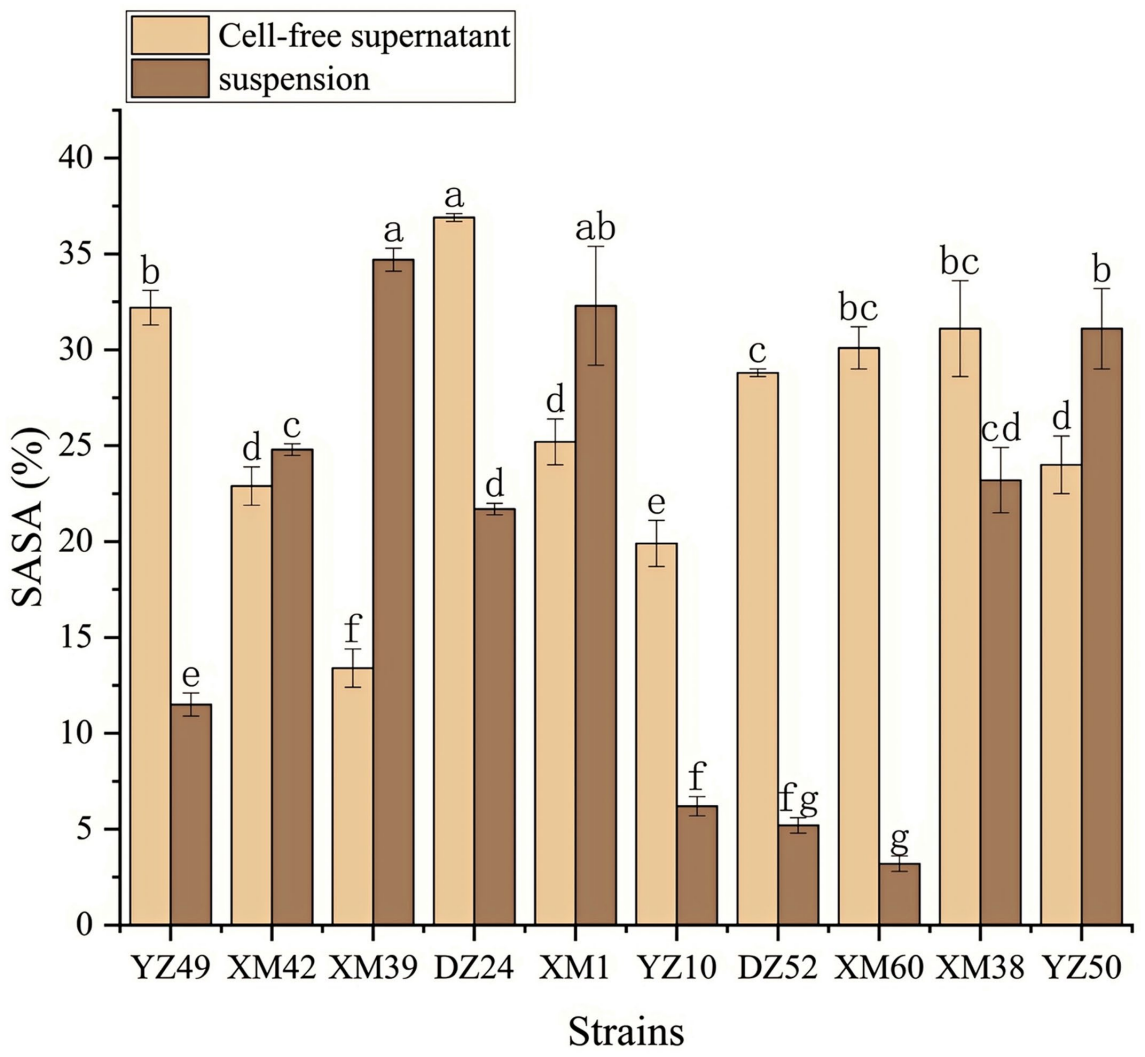
Superoxide anion radical scavenging rate of the 10 isolates. ^a–g^Values in the same row with different superscript letters are significantly different (*p* < 0.05).

**Figure 6:**
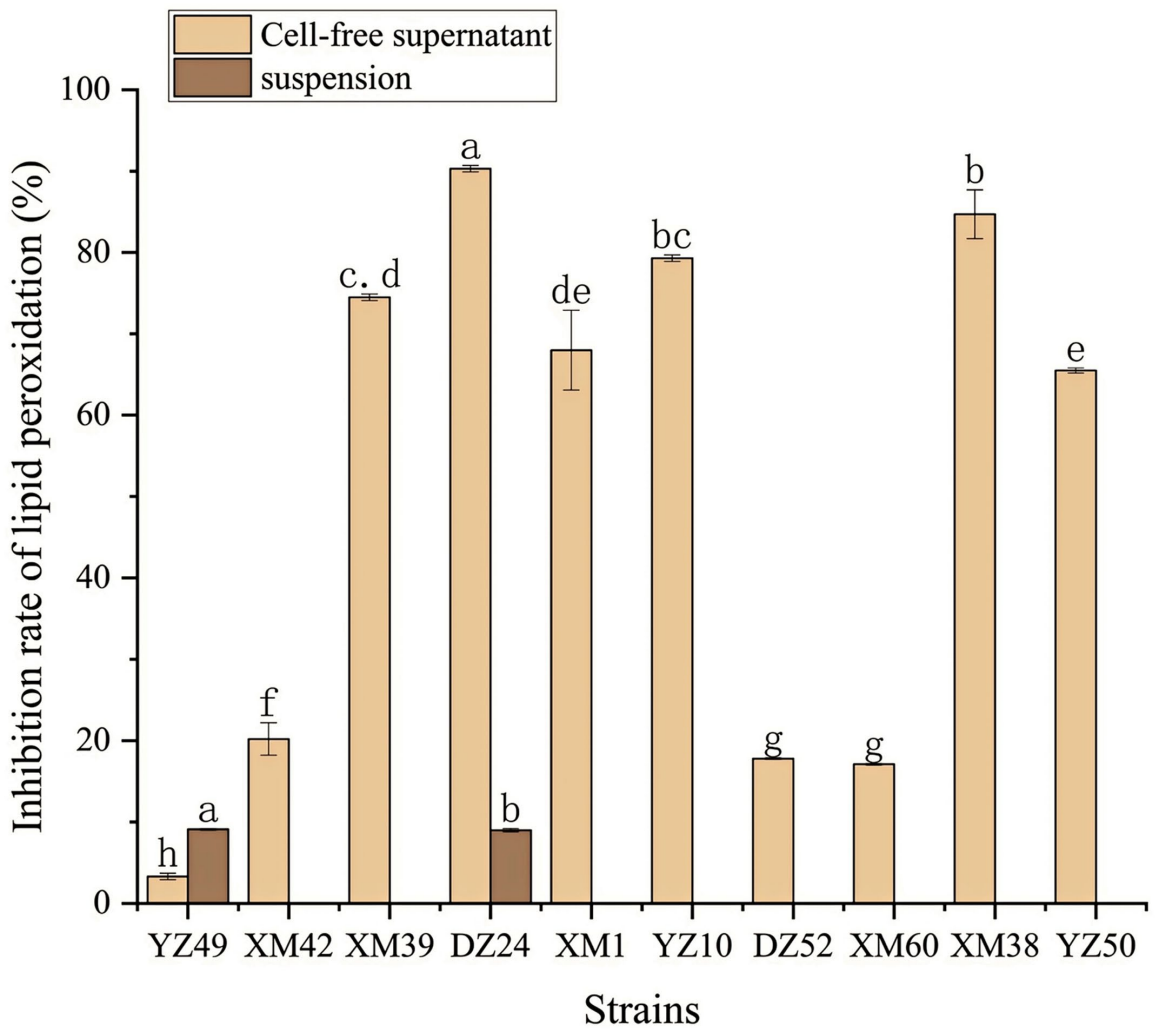
Anti-lipid-peroxidation capacity of the 10 isolates. ^a–g^Values in the same row with different superscript letters are significantly different (*p* < 0.05).

In human metabolism, the body spontaneously produces reactive oxygen species, such as hydroxyl radicals and superoxide anion radicals, which can destroy the balance between cell antioxidant capacity and the production of reactive oxygen species (Liu et al., 2021). This leads to oxidative stress, which may lead to diabetes, stroke, and other diseases (Liu et al., 2021). Therefore, the antioxidant activity of the strain is a very important probiotic property. As shown in Figures 4–6, all isolates exhibited different degrees of antioxidant ability. On the one hand, the DPPH radical scavenging rates of all isolates in the intact cell and cell-free groups were 5–58% and 13–34%, respectively (Figure 4). Among them, DZ24 was found with high antioxidant activity and the DPPH scavenging rates was 45.1%. Ji et al. (2015) reported that the DPPH scavenging rates of 11 *Lactobacillus* strains closed to 50%. Furthermore, *Lacticaseibacillus paracasei* (NM-11) isolated from fermented dairy products was a probiotic with high antioxidant activity and DPPH radical scavenging more than 30% (Abduxukur et al., 2023). The DPPH scavenging rate of DZ24 was 15% higher than that of this strain. Furthermore, Liu et al. (2020) reported the selected strains of LAB had a good DPPH scavenging ability of 42.6%. This was consistent with the results of this study. On the other hand, superoxide anion clearance of all isolates in the intact and cell-free groups ranged from 3 to 34.7% and 13–36.9%, respectively. The activity of XM39 was the highest in the intact cell group, accounting for 34.7%. The cell-free strain DZ24 had the highest superoxide anion-scavenging rate (36.9%) (Figure 5), which was higher than that of control Vitamin C (35.6%). Inhibition of lipid peroxidation is a well-established biomarker for evaluating the total antioxidant activity of bioactive ingredients in bacteria (Abduxukur et al., 2023). As shown in Figure 6, the cell-free supernatants of the 10 isolates all had a certain anti-lipid peroxidation ability, among which DZ24 had the highest value (90.3%). Chen et al. (2015) isolated strains from Harbin dry sausage in the intracellular cell-free extract; one of *P. pentosaceus* had the highest inhibitory effect on linoleic acid peroxidation with a rate of 57.1%. Besides, some studies have screened the LAB with excellent antioxidant activity from Tibetan mushrooms (Lee et al., 2020), and their superoxide anion scavenging rate and lipid peroxidation resistance reached 43.21 and 45.97%, respectively. Hence, these findings further support the better antioxidant activity observed in the DZ24 strain obtained in this study.

### 3.8 Molecular identification of LAB

Combining the results of the above experiments, 16S rRNA sequencing was performed on the best-performing DZ24, and the results were compared with the NCBI database. DZ24 was identified as *Lactiplantibacillus plantarum*, which provided important insights into the specific types of LAB that were isolated and their potential applications for further study and development (Table 3).

**Table 3:** Identification of 10 strains of LAB by 16S rRNA gene sequencing.

Strain	16sRNA molecular identification		
	Identity (%)	Identity	Access number^*^
DZ24	99.93%	*Lactiplantibacillus* *plantarum*	NR104573
YZ49	99.72%	*Pediococcus pentosaceus*	NR042058.1
XM1	–	*–*	–
XM42	–	*–*	–
XM39	–	*–*	–
XM60	–	*–*	–
XM38	99.93%	*Lactiplantibacillus* *plantarum*	NR115605.1
YZ5	99.13%	*Pediococcus stilesii*	NR042401.1
DZ52	99.58%	*Lactiplantibacillus* *plantarum*	NR117813
YZ10	99.79%	*Pediococcus* *acidilactici*	NR042057.1

^*^Number of sequences deposited at GenBank.

### 3.9 Results of fermentation characteristic test

The results presented in Table 4 indicated that *Lactiplantibacillus plantarum* DZ24 reduced the pH from 5.85 to 4.14 after 24 h of culture, demonstrating its rapid acid-production ability. Studies have shown that the acid-producing capacity of LAB is an important index to evaluate its fermentation characteristics. Similarly, Nie et al. (2023) also reported that the LAB (*Lactiplantibacillus plantarum*) reduced the pH from 5.75 to 4.50 after 24 h of culture. Moreover, *Lactiplantibacillus plantarum* DZ24 exhibited a tolerance level of 150 mg/kg nitrite under culture conditions. It was reported that the capacity to tolerate sodium nitrite concentrations was an important factor for probiotic viability in fermented meat products (Bis-Souza et al., 2020). A commercial starter culture was used in the fermentation of sausages showed that the starter culture could tolerate different concentrations of sodium chloride (1.5, 2.5, and 3%) and nitrite (100 and 150 ppm), conditions commonly used in the production of sausages (Mafra, 2020). Furthermore, the control medium was purple and the experimental medium was not purple, indicating that DZ24 does not contain amino acid deacylase. Besides, DZ24 could tolerate salt concentrations ranging from 20 to 80 g/L and retained a specific growth rate. Salinity adaptation studies of *Lactiplantibacillus plantarum* strains isolated from fermented foods at 20, 40, 60, and 80 g/L NaCl concentrations showed that the strain was able to grow at NaCl concentrations up to 80 g/L (Papadopoulou et al., 2023). The experimental results were in general agreement with the results of the present study.

**Table 4:** Technological characteristics of DZ24 strain.

Strain	Acid production (pH)			Nitrite tolerance^a^	Amino acid decarboxylase activity^b^	Salt tolerance (g/L)^a^			
	0 h	6 h	12 h	150 mg/kg		20	40	60	80
DZ24	5.85	4.89	4.14	+	–	+	+	+	+

a Bacterial growth: +; No growth: −.

b No purple color, negative: −.

## 4 Conclusion

Fermented sour meat was an excellent source for the isolation of LAB strains. There was significant microbial diversity in fermented sour meat from 4 different regions of China, in which *Lactobacillus* was the dominant genera. Among the 130 lactic acid bacteria isolates, only 10 strains of bacteria exhibited acid and bile salt resistance. Furthermore, *L. plantarum* DZ24 showed the most suitable properties as probiotics in terms of antimicrobial activity, cell surface hydrophobicity, antibacterial sensitivity and antioxidant activity. In addition, DZ24 meets the basic requirements for fermented meat products, including fast growth, fast acid production, and salt tolerance. This study revealed the probiotic potential of LAB strains from sour meat and provided potential probiotic candidates for fermented meat. However, *in vivo* experiments are needed to further assess its impact on public health.

## Data Availability

The data presented in the study are deposited in the NCBI repository, accession number: SAMN44789159, SAMN44789160, SAMN44789161, SAMN44789162.

## Supplementary material

Supplementary Figure S1 Microscopic examination of some strains after staining.

Supplementary Figure S2 Hemolysis test of the some strains.
