# Supplementary figures and images for: Isolation, identification, and evaluation of lactic acid bacteria with probiotic potential from traditional fermented sour meat
Source: Front Microbiol. 2024 Dec 12;15:1421285. doi: 10.3389/fmicb.2024.1421285 (PMC11669687; doi:10.3389/fmicb.2024.1421285)

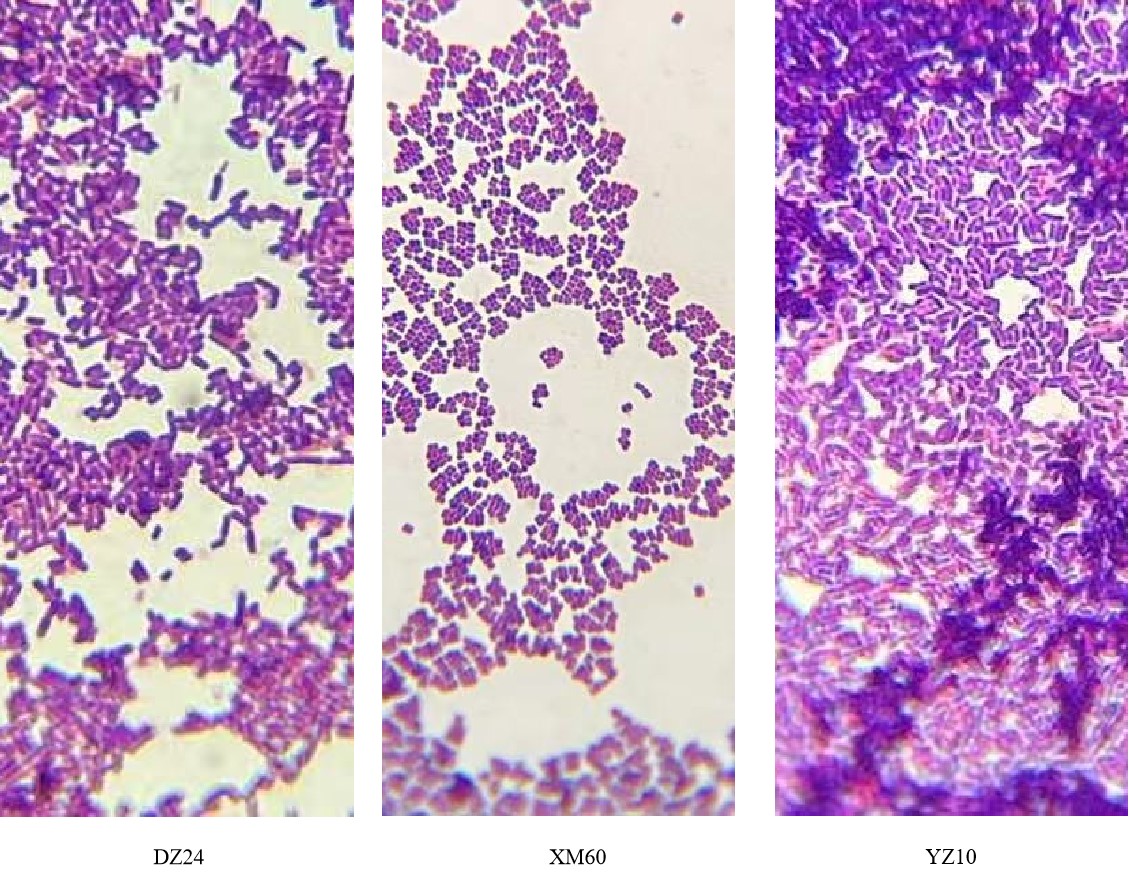

Supplement: Supplementary Figure S1 — Microscopic examination of some strains after staining. [file Image_1.jpeg]

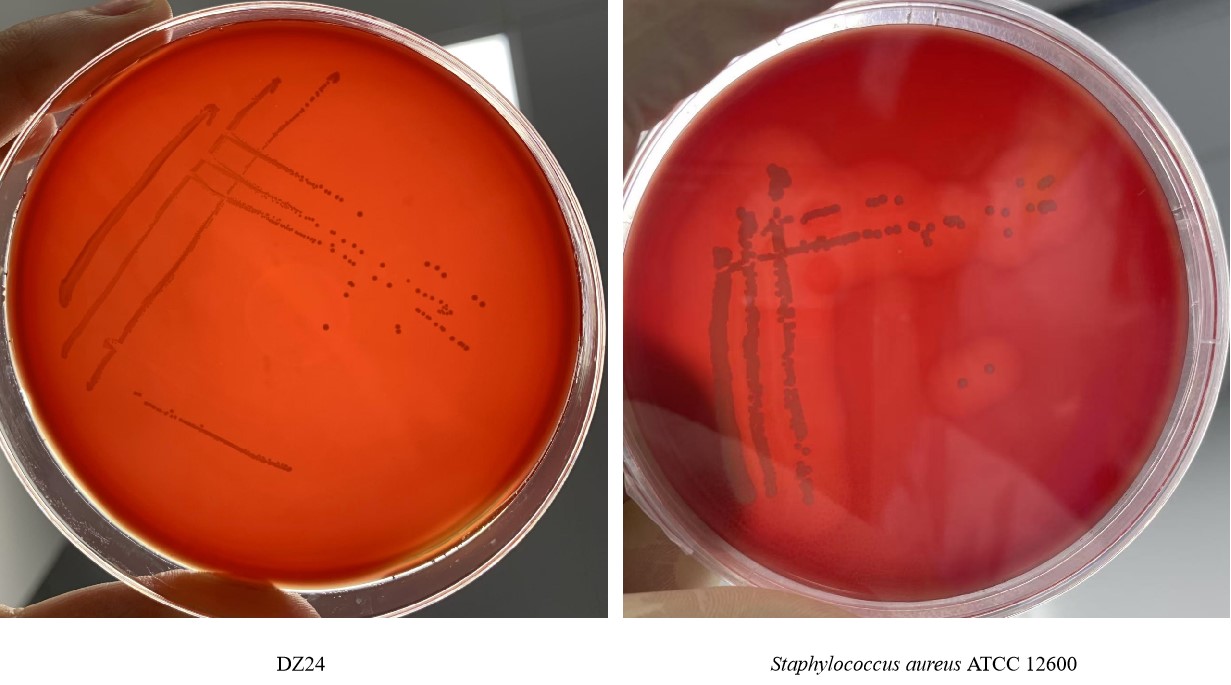

Supplement: Supplementary Figure S2 — Hemolysis test of the some strains. [file Image_2.jpeg]
